# Three years later: tracking bothersome symptoms and impacts for people with early Parkinson’s disease

**DOI:** 10.1007/s00415-026-13615-5

**Published:** 2026-01-21

**Authors:** Jennifer R. Mammen, Varun Reddy, Aaron Lerner, Nami Shah, Jocelyn Silva, Mirinda Tyo, Kristin Magan, Nguyen Thimaikhue, Dang Thao-Uyen, Tatiana Solodova, Tim McWilliams, Mathew Stephen, Peggy Auinger, Melissa Kostrzebski, Yuge Xiao, Diane Stephenson, Jamie L. Adams

**Affiliations:** 1 Dartmouth College of Nursing and Health Sciences, University of Massachusetts, 285 Old Westport Rd, Dartmouth, MA USA; 2 Center for Health + Technology, University of Rochester Medical Center, Rochester, NY USA; 3 Eastern Virginia Medical School, Norfolk, VA USA; 4 Department of Neurology, Medical Center, University of Rochester, Rochester, NY USA; 5 Michael J Fox Foundation for Parkinson’s Research, Newyork, USA; 6 Critical Path for Parkinson’s, Tucson, AZ USA

**Keywords:** Parkinson’s, Symptoms, Impacts, Longitudinal, Qualitative

## Abstract

**Background:**

Understanding how meaningful symptoms and impacts of Parkinson’s change with time is necessary to select endpoints for clinical trials and to support clinical practice.

**Objective:**

This study aimed to longitudinally evaluate the prevalence, bothersomeness, and functional impacts of early Parkinson’s symptoms on daily life over a three-year study duration.

**Methods:**

32 participants with early Parkinson’s completed qualitative interviews to map symptoms and impacts of disease annually for three years. Symptom maps were content coded for frequency and bothersomeness of symptoms and presence of related impacts. Non-parametric generalized linear mixed models (GLMM) were used to evaluate change over time.

**Results:**

The most bothersome motor symptoms were tremor, gait difficulties, balance, fine motor, slow movements, and stiffness at all years. Top non-motor symptoms were fatigue, sleep, mood changes, difficulty thinking, and quiet voice. Of these, only gait and balance changed significantly over the study duration. By contrast, many functional impacts changed significantly, with all reporting increased work of living and greater effort to do usual activities by year 3. At the same time, participants reported increased ability to cope and compensate by making positive life changes which mitigated the bothersomeness of symptoms.

**Conclusions:**

Other than gait and balance, few Parkinson’s symptoms increased significantly in bothersomeness over three years. However, functional and psychosocial impacts of symptoms, often attributed to more than one cause, were more sensitive to change over time.

**Supplementary Information:**

The online version contains supplementary material available at 10.1007/s00415-026-13615-5.

## Introduction

Determining which symptoms matter to people with Parkinson’s disease (PD) is needed for both clinical practice and to guide the selection of endpoints for clinical trials of disease modifying therapies [1–3]. Recent work in this area has identified a wide range of symptoms and impacts that are present and meaningful in early PD [4–8], however information on how these symptoms evolve with time remains quite limited [6, 9, 10]. This knowledge is necessary for clinicians providing clinical guidance to people with newly diagnosed Parkinson’s. It is also critical to researchers for selecting appropriate concepts of interest, endpoints for trials, optimal study length, and to quantify the elusive concept of “meaningful change” [2]. Thus, the purpose of this paper is to describe patient identified meaningful symptoms and impacts and how these changed with time in an early PD cohort that was followed for three years.

## Methods

**Fig. 1:**
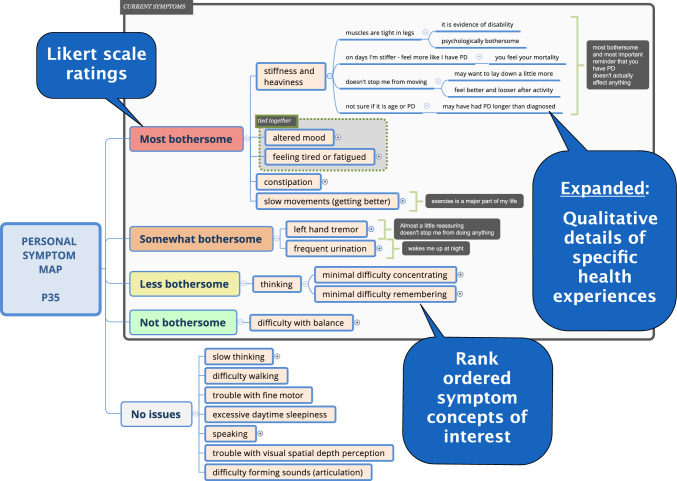
Symptom map structure

40 adults with early PD (criteria: < 2 years from diagnosis, Hoehn and Yahr (H&Y ≤ 2; no confounding co-morbidities) were randomly recruited from the WATCH-PD study [11] to participate in an online interview study (January 2021–July 2024) that used symptom mapping [12] to systematically identifying personally meaningful symptoms and impacts of disease and change over time. Each year, for three years, participants completed a brief online Redcap [13] survey comprising a comprehensive symptom checklist derived from the early PD conceptual model [14] and open-ended qualitative questions about current medication use, symptoms, and impacts of disease. Shortly following the survey, in-depth interviews were conducted online via Zoom using screen sharing and participants were asked to further describe each symptom they experienced, including specifics of how it affected them in daily life. Xmind software [15] was used to build a visual map of personal symptoms with dependent branching descriptions of symptom experiences as shown in Fig. 1. At the end of the interview, symptoms were reorganized by relative bothersomeness and rank ordered within the map to show which symptoms mattered most to each participant. Participants revised their map iteratively with the interviewer (JM) to reflect their personal experiences and validated all data entered on the map. PDF copies of the symptom map were given to the participant at the end of the interview for reciprocity and data validation.

### Data analysis

Completed symptom maps were coded using an Excel matrix to systematically identify prevalence and bothersomeness of all symptoms for each participant (Likert scale: Most bothersome = 4, Somewhat bothersome = 3, Less bothersome = 2, Not bothersome = 1, Not present = 0). Impacts for each symptom were coded as present or not present. Coding was performed independently by a first coder (VR), and all data was validated by a second coder (AL). Discrepancies were resolved by arbitration with other team members (MT/JS) to achieved 100% consensus. Using SPSS29, symptom frequencies were assessed by year using descriptive statistics, and non-parametric generalized linear mixed models (GLMM) were used to evaluate change in symptoms and impacts over time.

### Ethical statement

IRB approval (University of Rochester IRB#00006429; University of Massachusetts, Dartmouth IRB#23.044) and electronic informed consent were obtained prior to data collection.

## Results

### Demographics

32 participants completed all three years of symptom mapping interviews (80% retention) for a total of 96 interviews. Interview duration averaged 2 h in length per person, per year. Of the original 40 people recruited, four were lost to contact due to IRB protocol transition issues after year 1 and four declined to continue longitudinally due to lack of time. As presented in Table 1, mean age at baseline was 63 years, with 95% being white and 56% being male. Montreal Cognitive Assessment (MoCA) remained consistent across years, with an average score of 28 (normal cognition). H&Y gradually increased over time. However, 87.5% of participants maintained H&Y ≤ 2 by year 3. At years 2 and 3, respectively, 75–78% of participants reported taking PD medications (e.g., carbidopa/levodopa, rasagiline, amantadine) and one reported deep brain stimulation (DBS) as compared to only 40% using PD medications at year 1. There were no significant demographic differences between the WATCH-PD parent studies or between interview completers and non-completers.

### Bothersome symptoms of Parkinson’s disease

**Fig. 2:**
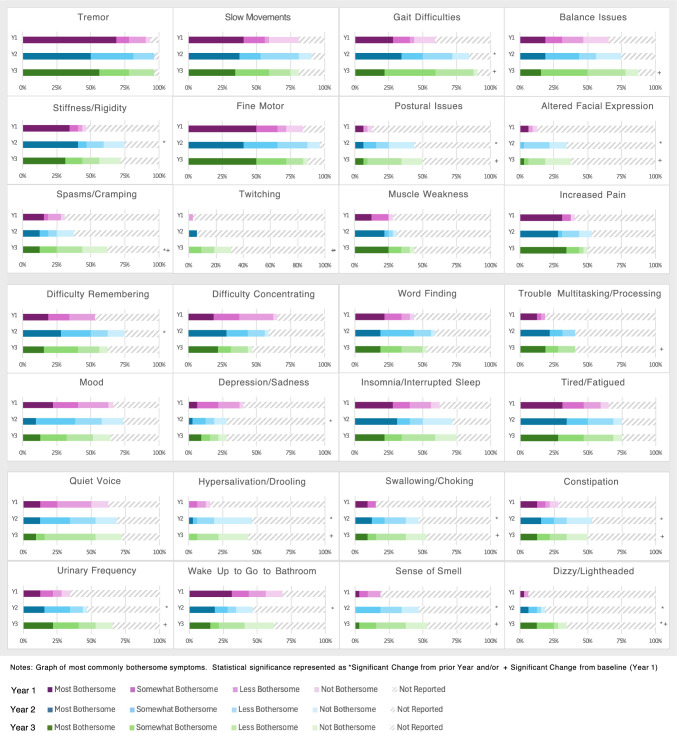
Most bothersome symptoms of early Parkinson's over three years

Relative frequencies and bothersomeness of top motor and non-motor symptoms across three years are presented in Fig. 2 with frequencies of all symptoms reported by year in online **supplementary tables**. Of motor symptoms reported at year 3, tremor (97%), gait difficulties (90%), balance issues (87%), fine motor (87%), slow movements (81%), and stiffness (71%) were the most reported bothersome symptoms. Comparing baseline to year 3, only gait difficulties (59.4% vs 90.6%, *p* < 0.01) and balance issues (65.6% vs 87.5% *p* < 0.01) became significantly more bothersome with time. Other less common motor symptoms that changed significantly from baseline to year 3 included spasms/cramping (31.2% vs 62.5%, *p* < 0.05), postural issues (12.5% vs. 50%, *p* < 0.01), altered facial expression (12.5% vs. 37.5% *p* < 0.01), and twitching (3.1% vs. 31.2%, *p* < 0.05).

The top non-motor symptoms at year 3 were fatigue, sleep problems, non-specific mood changes, difficulty thinking, and quiet voice, all of which were generally stable across years. Notably, participants tended to report less depression over time (41% year 1 vs. 29% year 3), whereas anxiety remained constant around 50% for all years (**Supplementary Tables**). Comparing year 1 to year 3, urinary urgency/frequency increased significantly (34.4% vs 65.6%, *p* < 0.01) as did urinary incontinence (0% vs 15.60%, *p* < 0.001). Other significant changes were trouble multitasking/processing (18.7% vs 40.6%, *p* < 0.05), feeling dizzy or lightheaded (6.2% vs 34.4%, *p* < 0.01), diminished sense of smell (18.7% vs 53.1%, *p* < 0.01), and reduced taste (0% vs 31.2%, *p* < 0.001). Multiple changes were identified in the digestive domain, including increased issues with swallowing/choking (15.6% vs 53.1%, *p* < 0.01), constipation (28.1% vs 50%, *p* < 0.05), drooling (15.6% vs 43.7%, *p* < 0.05), and loss of appetite (3.1% vs 18.7%, *p* < 0.05). With regard to less-recognized symptoms at year 3, many reported pain (34.4%) and muscle weakness (25%) as among the most bothersome PD symptoms, however, without significant change from baseline.

### Physical and psychosocial impacts of symptoms

**Fig. 3:**
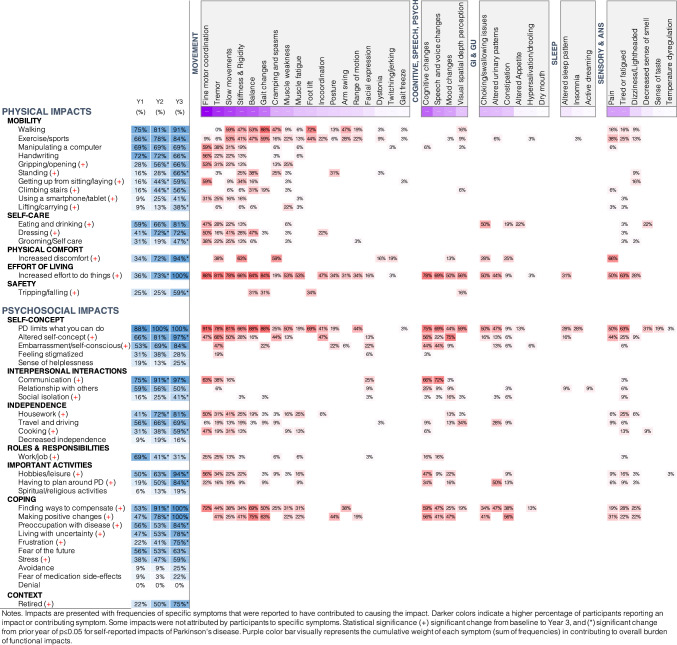
Physical and psychosocial impacts of early Parkinson's over three years

All participants reported that PD impacted daily functioning, with statistically significant differences observed in multiple areas, as shown in Fig. 3. Fine motor, tremor, slow movements, cognitive changes, stiffness, and gait/balance were the most reported causes of functional impacts, and all participants reported increased effort to do things by year 3 as PD progressed, which was a significant increase from 36% in year 1. By year 3, 75% of participants had retired, and fewer people reported job-related impacts (69% Y1 vs. 31% Y3).

***P35:**** Lack of dexterity [affects] typing…and texting…It makes me feel more debilitated than I am, because I can still do things like run and box and I'm completely independent, but when I see my hands having such a hard time, it's psychologically very hard. (Female, age 66)*

At year 3, most common psychosocial impacts included greater difficulty communicating (97%), altered self-concept (97%), embarrassment (84%) increased trouble with household activities (81%), decreased ability to engage in hobbies and pleasurable activities (81%), and driving limitations (69%), all of which increased significantly from baseline (*p* < 0.05). Other significant and pervasive psychosocial impacts included greater preoccupation with disease, having to plan around Parkinson’s symptoms, increased frustration, fear of what the future will bring, and greater personal distress (Fig. 3). Less than half of participants reported social isolation. However, isolation increased significantly over time (16% year 1 vs. 41% year 3, *p* < 0.05).

***P10****: Sometimes I'll start talking and the words will get garbled… I spend more time searching for a specific word…. [and] my speech isn't as fluid. It affects my enjoyment of conversation. …My social life is decreasing. I'm making efforts to keep it going, but it really feels like an effort. (Female, age 63)*

Comparing baseline to year 3, highly significant physical impacts included greater effort to do usual activities (36% vs. 100%, *p* < 0.001) and greater physical discomfort (34% at year 1 increasing to 94% by year 3, *p* < 0.001), most commonly affecting walking and exercise, as well as increased trouble with gripping/opening (28% vs 66%, *p* < 0.01), standing (16% vs 66%, *p* < 0.001), getting up (16% vs 59%, *p* < 0.001), climbing stairs (16% vs 56%, *p* < 0.01), tripping/falling (18.7% vs. 46.9%, *p* < 0.01), eating/drinking (59% vs. 81%, *p* < 0.05), and dressing (41% vs. 72%, *p* < 0.01).

***P6:**** I am reducing activities [because of fatigue and stiffness]. I have to adjust how much energy I expend. If I'm gonna get up and go from the sofa and go to the kitchen, I now ask myself, is this worth doing it? (Female, age 62)*

***P15:**** [I have trouble] getting out of bed [and] standing up [because of pain and stiffness]. I purchased a lift chair for myself to facilitate that…It takes me way longer to dress than it should. I still do the grocery shopping with my wife [and we] occasionally go out to lunch with other people. It’s not stopping me from doing anything. It’s just slowing me down. (Male, age 82)*

Despite the escalating functional impact of disease, by year 3 all participants were making positive changes to take control and manage PD symptoms, such as exercising, meditating, stress management, and engaging in meaningful activities, which served to mitigate the impact and bothersomeness of symptoms. Participants more commonly discussed the effectiveness of cognitive and behavioral strategies for symptom control (84% engaging, average 6 mentions/interview, range 0–17) than medication use (72% taking, average 3 mentions/interview, Range 0–10, *p* < 0.01).

***P13****: Everything in my body is moving so slow. My decision making is slow. [But] I'm learning how to deal with my body [and] I don't feel as uncomfortable now. I just adjust [and] I try to do more positive thinking. (Male, age 72)*

## Discussion

This is the first longitudinal mixed-methods study of what is important to people in early Parkinson’s disease, contributing to a deeper understanding of symptom experiences and meaningful changes from the patient perspective. Like prior studies, our findings suggest that non-motor symptoms of fatigue, cognitive changes, sleep disturbances, urinary symptoms, and altered voice, along with motor symptoms of tremor, balance, gait, fine motor, slow movements, and stiffness, were consistently most important to patients [14, 16, 17]. However, it is important to note that only gait, balance, and posture were rated as more bothersome within three years. In contrast to most symptoms, which remained stably reported in frequency, participant’s reports of functional and psychosocial impacts secondary to symptoms increased substantially, with psychosocial impacts (e.g., self-concept) far outpacing functional impacts. The slower self-perceived change in symptoms compared to the more rapid evolution of impacts may be reflective of the multifocal nature of impacts (i.e., impacts being attributable to several symptoms conjointly), gradual disease progression, effectiveness of new medications, and greater knowledge and capacity to adapt in early PD.

These findings of statistically non-significant patient-perceived change in most symptoms over three years has distinct implications for clinical trials, suggesting that standard patient reported outcome measures may fail to capture early disease progression within a 12- to 18-month clinical trial timeframe, which is consistent with prior reports [18]. Digital monitoring technologies, by contrast, have shown promise as sensitive and time efficient alternatives to measure early symptom progression [11, 19], but with currently limited evidence, which is mostly single time-point, to connect digital-data to lived-experiences [20, 21]. Findings from our study combined with prior evidence suggest that digital measures of gait and balance combined with repeated patient reported measures focusing on mobility-related impacts might be an effective means to connect digital findings with lived-experiences and identify thresholds for meaningful change. Further work is needed to define endpoints for early PD, thresholds for meaningful change, and optimal duration for clinical trials of disease modifying treatments in early PD.

From a clinical standpoint, educating patients on positive lifestyle changes and stress management strategies could substantially mitigate the personal impact of symptoms in early PD, help to improving coping, and empower patients to take control of disease progression. Resources, such as Rock-Steady Boxing, peer support groups, and meditation, were commonly referenced coping aids in our cohort, with good evidence of efficacy found in prior literature [22–25]. Based on these findings, we would conclude that early interventions to support coping and adaptation are likely to be mentally and physically beneficial and lead to better patient outcomes.

Limitations of this study include a smaller sample size with predominantly white participants. Further work will be needed to determine applicability of findings to racially diverse populations and to explore possible sex-based differences. Additionally, due to study design, we were unable to fully assess the range of coping strategies and patient-perceived benefits for managing different symptoms. Future research in this area is likely to benefit patients and families seeking non-pharmacologic means to manage symptoms. Finally, this study focused on bothersomeness of symptoms (i.e., personal relevance to the patient) rather than symptom severity, which is a more clinically oriented concept. Thus, it is possible that symptoms could have increased from a clinical perspective yet did not change in a way patients perceived to be personally meaningful. Objective comparison of symptom severity (digital or clinician rated) with subjective measures of patient-perceived severity and bothersomeness may help connect and interpret observed versus experienced symptoms and define meaningful change for early PD.

## Electronic supplementary material

Below is the link to the electronic supplementary material.

Supplementary file1 (PDF 125 KB)

## Data Availability

De-identified datasets are available upon request.

## Appendix A

See Table 1.

**Table 1:** Demographic and clinical characteristics

	**Year 1 interview** **(*****n***** = 32)**	**Year 2 interview** **(*****n***** = 32)**	**Year 3 interview** **(*****n***** = 32)**
Age, years, mean (SD)	64.4 (8.9)	65.9 (8.8)	66.8 (9.4)
Female, *n* (%)	14 (43.8%)	14 (43.8%)	14 (43.8%)
Race/ethnicity, *n* (%)			
White	29 (90.6%)	29 (90.6%)	29 (90.6%)
Asian	3 (9.4%)	3 (9.4%)	3 (9.4%)
Not specified	−	−	−
Hispanic or Latino, *n* (%)	0 (0%)	0 (0%)	0 (0%)
Education > 12 years, *n* (%)	32 (100%)	32 (100%)	32 (100%)
PD duration, years, mean (SD)	2.2 (0.9)	3.2 (0.7)	4.5 (0.9)
Taking medications for PD, *n* (%)	13 (40.6%)	24 (75.0%)	25 (78.1%)^a^
H&Y, *n* (%)			
Stage 1	4 (12.5%)	4 (12.5%)	0 (0%)
Stage 2	28 (87.5%)	24 (75.0%)	28 (87.5%)
Stage 3	−	2 (6.3%)	1 (3.1%)
Stage 4	−	−	1 (3.1%)
Not specified	−	2 (6.3%)	2 (6.3%)
MoCA	28.0 (2.4)	27.0 (2.2)	28.0 (2.5)

*MoCA* montreal cognitive assessment scores of 26–30 are considered normal, *H&Y* hoehn & yahr stage ≤ 2 is consistent with early PD

^a^Includes one participant on PD medication plus treated with Deep Brain Stimulation

## Declarations

### Quality in qualitative research statement

This study used COREQ criteria to guide reporting of qualitative findings.
