# Supplementary material for: Three years later: tracking bothersome symptoms and impacts for people with early Parkinson’s disease
Source: J Neurol. 2026 Jan 21;273(2):93. doi: 10.1007/s00415-026-13615-5 (PMC12823644; doi:10.1007/s00415-026-13615-5)
Supplement: Supplementary file 1 — Supplementary file1 (PDF 125 KB) [file 415_2026_13615_MOESM1_ESM.pdf]

Appendix A. Symptom Frequency Tables

Movement Domain

Year 1 Movement Domain

Year 1 Symptoms	Most Bothersome	Somewhat Bothersome	Less Bothersome	Not Bothersome	Not Reported
Stiffness or Rigidity	34.40%	6.30%	3.10%	3.10%	53.10%
Spasms, Cramping, Dystonia, or Contractures	15.60%	3.10%	9.40%	3.10%	68.80%
Slow Movements	40.60%	15.60%	3.10%	21.90%	18.80%
Gait Difficulties or Changes	28.10%	12.50%	3.10%	15.60%	40.60%
Balance Issues	18.80%	12.50%	15.60%	18.80%	34.40%
Altered Arm Swing	18.80%	9.40%	3.10%	6.30%	62.50%
Postural Issues	6.30%	0.00%	3.10%	3.10%	87.50%
Decreased Range of Motion	18.80%	12.50%	3.10%	3.10%	62.50%
Altered Facial Expression	6.30%	0.00%	3.10%	3.10%	87.50%
Dyskinesias	0.00%	0.00%	0.00%	0.00%	100.00%
General Incoordination or Gross Motor Control	16.10%	6.50%	0.00%	3.20%	74.20%
Freezing	0.00%	0.00%	0.00%	0.00%	100.00%
Restless Legs Syndrome	0.00%	0.00%	3.20%	0.00%	96.80%
Twitching	0.00%	0.00%	3.10%	0.00%	96.90%
Any tremor	68.80%	9.40%	12.50%	3.10%	6.30%
Hand or Arm Tremor	56.30%	9.40%	12.50%	3.10%	18.80%
Leg or Foot Tremor	28.10%	0.00%	9.40%	3.10%	59.40%
Jaw, Neck, Face, or Other Tremor	9.40%	0.00%	0.00%	0.00%	90.60%
Sense of Internal Tremor	0.00%	0.00%	3.10%	0.00%	96.90%
Fine Motor Category	50.00%	15.60%	6.30%	12.50%	15.60%
Finger Coordination	43.80%	6.30%	3.10%	3.10%	43.80%

Year 2 Movement Domain

Year 2 Symptoms	Most Bothersome	Somewhat Bothersome	Less Bothersome	Not Bothersome	Not Reported
Stiffness or Rigidity*	40.60%	6.30%	12.50%	15.60%	25.00%
Spasms, Cramping, Dystonia, or Contractures	12.50%	6.30%	6.30%	12.50%	62.50%
Slow Movements	37.50%	15.60%	28.10%	9.40%	9.40%
Gait Difficulties or Changes*	34.40%	15.60%	21.90%	12.50%	15.60%
Balance Issues	18.80%	25.00%	12.50%	18.80%	25.00%
Altered Arm Swing	9.40%	6.30%	9.40%	12.50%	62.50%
Postural Issues*	6.30%	9.40%	9.40%	18.80%	56.30%
Decreased Range of Motion	15.60%	3.10%	6.30%	0.00%	75.00%
Altered Facial Expression*	0.00%	3.10%	18.80%	12.50%	65.60%
Dyskinesias	15.60%	0.00%	0.00%	0.00%	84.40%
General Incoordination or Gross Motor Control	28.10%	6.30%	3.10%	0.00%	62.50%
Freezing	0.00%	0.00%	0.00%	0.00%	100.00%
Restless Legs Syndrome	6.30%	0.00%	0.00%	0.00%	93.80%
Twitching	6.30%	0.00%	0.00%	0.00%	93.80%
Any tremor	50.00%	31.30%	15.60%	0.00%	3.10%
Hand or Arm Tremor	43.80%	25.00%	12.50%	0.00%	18.80%
Leg or Foot Tremor	25.00%	3.10%	9.40%	0.00%	62.50%
Jaw, Neck, Face, or Other Tremor	6.30%	6.30%	0.00%	0.00%	87.50%
Sense of Internal Tremor	3.10%	3.10%	0.00%	0.00%	93.80%
Fine Motor Category	40.60%	25.00%	21.90%	9.40%	3.10%
Finger Coordination	31.30%	15.60%	9.40%	0.00%	43.80%

Year 3 Movement Domain

Year 3 Symptoms	Most Bothersome	Somewhat Bothersome	Less Bothersome	Not Bothersome	Not Reported
Stiffness or Rigidity	31.30%	12.50%	12.50%	15.60%	28.10%
Spasms, Cramping, Dystonia, or Contractures+*	12.50%	12.50%	18.80%	18.80%	37.50%
Slow Movements	34.40%	25.00%	15.60%	6.30%	18.80%
Gait Difficulties or Changes+	21.90%	37.50%	28.10%	3.10%	9.40%
Balance Issues+	15.60%	34.40%	28.10%	9.40%	12.50%
Altered Arm Swing	6.30%	15.60%	12.50%	25.00%	40.60%
Postural Issues+	6.30%	3.10%	25.00%	15.60%	50.00%
Decreased Range of Motion	18.80%	6.30%	6.30%	3.10%	65.60%
Altered Facial Expression+	3.10%	3.10%	12.50%	18.80%	62.50%
Dyskinesias**+**	3.10%	3.10%	0.00%	0.00%	93.80%
General Incoordination or Gross Motor Control	28.10%	3.10%	9.40%	0.00%	59.40%
Freezing	3.10%	3.10%	0.00%	0.00%	93.80%
Restless Legs Syndrome	0.00%	3.10%	3.10%	0.00%	93.80%
Twitching**+***	0.00%	9.40%	9.40%	12.50%	68.80%
Any tremor	56.30%	21.90%	18.80%	0.00%	3.10%
Hand or Arm Tremor	53.10%	18.80%	18.80%	0.00%	9.40%
Leg or Foot Tremor	18.80%	6.30%	12.50%	0.00%	62.50%
Jaw, Neck, Face, or Other Tremor	9.40%	3.10%	6.30%	0.00%	81.30%
Sense of Internal Tremor	3.10%	0.00%	3.10%	0.00%	93.80%
Fine Motor Category	50.00%	21.90%	12.50%	3.10%	12.50%
Finger Coordination	43.80%	18.80%	12.50%	3.10%	21.90%

*Significant Change from prior Year
+Significant Change from baseline(Year 1)

Speech & Voice Domain

Year 1 Speech & Voice Domain

Year 1 Symptoms	Most Bothersome	Somewhat Bothersome	Less Bothersome	Not Bothersome	Not Reported
Quiet Voice	12.50%	12.50%	25.00%	12.50%	37.50%
Articulation	6.30%	12.50%	15.60%	9.40%	56.30%
Monotone Voice	9.40%	0.00%	12.50%	6.30%	71.90%
Change in Vocal Quality	9.40%	9.40%	6.30%	0.00%	75.00%

Year 2 Speech & Voice Domain

Year 2 Symptoms	Most Bothersome	Somewhat Bothersome	Less Bothersome	Not Bothersome	Not Reported
Quiet Voice	12.50%	21.90%	18.80%	15.60%	31.30%
Articulation	9.40%	18.80%	9.40%	9.40%	53.10%
Monotone Voice	9.40%	9.40%	12.50%	6.30%	62.50%
Change in Vocal Quality*	12.50%	21.90%	15.60%	6.30%	43.80%

Year 3 Speech & Voice Domain

Year 3 Symptoms	Most Bothersome	Somewhat Bothersome	Less Bothersome	Not Bothersome	Not Reported
Quiet Voice	9.40%	6.30%	37.50%	18.80%	28.10%
Articulation	9.40%	12.50%	21.90%	6.30%	50.00%
Monotone Voice	3.10%	3.10%	21.90%	12.50%	59.40%
Change in Vocal Quality	9.40%	3.10%	21.90%	15.60%	50.00%

*Significant Change from prior Year
+Significant Change from baseline(Year 1)

Psychiatric Domain

Year 1 Psychiatric Domain

Year 1 Symptoms	Most Bothersome	Somewhat Bothersome	Less Bothersome	Not Bothersome	Not Reported
Mood	22.20%	18.50%	22.20%	3.70%	33.30%
Anxiety	15.60%	15.60%	18.80%	0.00%	50.00%
Depression or Sadness	6.30%	15.60%	15.60%	3.10%	59.40%
Apathy	6.30%	3.10%	3.10%	0.00%	87.50%
More Emotional	0.00%	0.00%	3.10%	0.00%	96.90%
Irritability	3.10%	0.00%	6.30%	0.00%	90.60%
Personality Changes	0.00%	0.00%	0.00%	0.00%	100.00%
Psychosis	0.00%	0.00%	0.00%	0.00%	100.00%
Flat Mood or Disinterested	3.10%	0.00%	0.00%	0.00%	96.90%

Year 2 Psychiatric Domain

Year 2 Symptoms	Most Bothersome	Somewhat Bothersome	Less Bothersome	Not Bothersome	Not Reported
Mood	9.70%	29.00%	19.40%	16.10%	25.80%
Anxiety	12.50%	12.50%	9.40%	12.50%	53.10%
Depression or Sadness*	3.10%	9.40%	6.30%	9.40%	71.90%
Apathy	0.00%	15.60%	9.40%	6.30%	68.80%
More Emotional	9.40%	0.00%	0.00%	0.00%	90.60%
Irritability	6.30%	9.40%	9.40%	3.10%	71.90%
Personality Changes	0.00%	15.60%	9.40%	0.00%	75.00%
Psychosis	3.20%	0.00%	0.00%	0.00%	96.80%
Flat Mood or Disinterested	0.00%	0.00%	3.20%	0.00%	96.80%

Year 3 Psychiatric Domain

Year 3 Symptoms	Most Bothersome	Somewhat Bothersome	Less Bothersome	Not Bothersome	Not Reported
Mood	12.90%	19.40%	19.40%	12.90%	35.50%
Anxiety	12.50%	9.40%	15.60%	9.40%	53.10%
Depression or Sadness	9.40%	6.30%	6.30%	6.30%	71.90%
Apathy	9.40%	0.00%	12.50%	6.30%	71.90%
More Emotional	3.10%	3.10%	0.00%	0.00%	93.80%
Irritability	3.10%	9.40%	15.60%	0.00%	71.90%
Personality Changes+	0.00%	6.30%	6.30%	6.30%	81.30%
Psychosis+	0.00%	0.00%	3.10%	3.10%	93.80%
Flat Mood or Disinterested	0.00%	6.30%	3.10%	3.10%	87.50%

*Significant Change from prior Year

+Significant Change from baseline(Year 1)

Sleep Domain

Year 1 Sleep Domain

Year 1 Symptoms	Most Bothersome	Somewhat Bothersome	Less Bothersome	Not Bothersome	Not Reported
Insomnia or Interrupted Sleep	28.10%	12.50%	15.60%	6.30%	37.50%
Daytime Sleepiness	18.80%	9.40%	3.10%	3.10%	65.60%
Acting Out Dreams or Vivid Dreaming	12.50%	12.50%	3.10%	3.10%	68.80%

Year 2 Sleep Domain

Year 2 Symptoms	Most Bothersome	Somewhat Bothersome	Less Bothersome	Not Bothersome	Not Reported
Insomnia or Interrupted Sleep	31.30%	9.40%	9.40%	21.90%	28.10%
Daytime Sleepiness	18.80%	9.40%	0.00%	3.10%	68.80%
Acting Out Dreams or Vivid Dreaming	12.50%	6.30%	3.10%	6.30%	71.90%

Year 3 Sleep Domain

Year 3 Symptoms	Most Bothersome	Somewhat Bothersome	Less Bothersome	Not Bothersome	Not Reported
Insomnia or Interrupted Sleep	21.90%	12.50%	25.00%	15.60%	25.00%
Daytime Sleepiness	12.50%	9.40%	12.50%	9.40%	56.30%
Acting Out Dreams or Vivid Dreaming	12.50%	0.00%	12.50%	15.60%	59.40%

*Significant Change from prior Year
+Significant Change from baseline(Year 1)

Cognitive Domain

Year 1 Cognitive Domain

Year 1 Symptoms	Most Bothersome	Somewhat Bothersome	Less Bothersome	Not Bothersome	Not Reported
Difficulty Concentrating	18.80%	18.80%	25.00%	3.10%	34.40%
Slower Thinking	25.00%	12.50%	18.80%	0.00%	43.80%
Difficulty Remembering	18.80%	15.60%	18.80%	0.00%	46.90%
Word Finding Issues	21.90%	12.50%	6.30%	3.10%	56.30%
Left and Right Confusion	0.00%	3.10%	0.00%	0.00%	96.90%
Visual Spatial Depth Perception	6.30%	9.40%	6.30%	9.40%	68.80%
Trouble Multitasking or Processing	12.50%	3.10%	3.10%	0.00%	81.30%

Year 2 Cognitive Domain

Year 2 Symptoms	Most Bothersome	Somewhat Bothersome	Less Bothersome	Not Bothersome	Not Reported
Difficulty Concentrating	28.10%	15.60%	12.50%	3.10%	40.60%
Slower Thinking	25.00%	21.90%	15.60%	3.10%	34.40%
Difficulty Remembering*	28.10%	21.90%	12.50%	12.50%	25.00%
Word Finding Issues	18.80%	25.00%	12.50%	3.10%	40.60%
Left and Right Confusion	0.00%	0.00%	0.00%	0.00%	100.00%
Visual Spatial Depth Perception	6.30%	12.50%	15.60%	9.40%	56.30%
Trouble Multitasking or Processing	21.90%	9.40%	9.40%	0.00%	59.40%

Year 3 Cognitive Domain

Year 3 Symptoms	Most Bothersome	Somewhat Bothersome	Less Bothersome	Not Bothersome	Not Reported
Difficulty Concentrating	21.90%	9.40%	12.50%	3.10%	53.10%
Slower Thinking	21.90%	25.00%	15.60%	3.10%	34.40%
Difficulty Remembering	15.60%	25.00%	15.60%	6.30%	37.50%
Word Finding Issues	18.80%	15.60%	15.60%	3.10%	46.90%
Left and Right Confusion	0.00%	0.00%	0.00%	0.00%	100.00%
Visual Spatial Depth Perception	6.30%	6.30%	25.00%	15.60%	46.90%
Trouble Multitasking or Processing+	18.80%	9.40%	12.50%	0.00%	59.40%

*Significant Change from prior Year
+Significant Change from baseline(Year 1)

Autonomic Domain

Year 1 Autonomic Domain

Year 1 Symptoms	Most Bothersome	Somewhat Bothersome	Less Bothersome	Not Bothersome	Not Reported
Feeling Dizzy or Lightheaded	3.10%	0.00%	3.10%	0.00%	93.80%
Temperature Dysregulation	3.10%	0.00%	0.00%	0.00%	96.90%
Blood Pressure Issues	0.00%	0.00%	0.00%	0.00%	100.00%
Lower Leg Swelling	0.00%	0.00%	0.00%	0.00%	100.00%

Year 2 Autonomic Domain

Year 2 Symptoms	Most Bothersome	Somewhat Bothersome	Less Bothersome	Not Bothersome	Not Reported
Feeling Dizzy or Lightheaded*	6.30%	6.30%	3.10%	3.10%	81.30%
Temperature Dysregulation	0.00%	0.00%	0.00%	0.00%	100.00%
Blood Pressure Issues	0.00%	6.30%	0.00%	0.00%	93.80%
Lower Leg Swelling	0.00%	0.00%	0.00%	0.00%	100.00%

Year 3 Autonomic Domain

Year 3 Symptoms	Most Bothersome	Somewhat Bothersome	Less Bothersome	Not Bothersome	Not Reported
Feeling Dizzy or Lightheaded+*	12.50%	12.50%	3.10%	6.30%	65.60%
Temperature Dysregulation	3.10%	0.00%	0.00%	0.00%	96.90%
Blood Pressure Issues+	6.30%	6.30%	0.00%	0.00%	87.50%
Lower Leg Swelling	0.00%	0.00%	0.00%	0.00%	100.00%

*Significant Change from prior Year
+Significant Change from baseline(Year 1)

Sensation Domain

Year 1 Sensory Domain

Year 1 Symptoms	Most Bothersome	Somewhat Bothersome	Less Bothersome	Not Bothersome	Not Reported
Increased Pain	31.30%	6.30%	0.00%	3.10%	59.40%
Diminished Sense of Smell	3.10%	6.30%	9.40%	0.00%	81.30%
Diminished Sensation	3.10%	0.00%	0.00%	0.00%	96.90%
Double Vision or Vision Changes	0.00%	0.00%	0.00%	0.00%	100.00%
Dry Eyes	0.00%	0.00%	0.00%	0.00%	100.00%
Tearing of Eyes	0.00%	0.00%	3.10%	0.00%	96.90%
Headaches	0.00%	0.00%	0.00%	0.00%	100.00%
Numbness and Tingling	0.00%	0.00%	0.00%	0.00%	100.00%
Altered Sense of Taste	0.00%	0.00%	0.00%	0.00%	100.00%
Muscle Weakness	12.50%	12.50%	0.00%	3.10%	71.90%
Muscle Fatigue	18.80%	3.10%	0.00%	0.00%	78.10%
Tired or Fatigued	31.30%	15.60%	12.50%	6.30%	34.40%

Year 2 Sensory Domain

Year 2 Symptoms	Most Bothersome	Somewhat Bothersome	Less Bothersome	Not Bothersome	Not Reported
Increased Pain	28.10%	3.10%	12.50%	9.40%	46.90%
Diminished Sense of Smell*	0.00%	18.80%	15.60%	12.50%	53.10%
Diminished Sensation	3.10%	3.10%	6.30%	0.00%	87.50%
Double Vision or Vision Changes	0.00%	0.00%	0.00%	0.00%	100.00%
Dry Eyes	3.10%	0.00%	0.00%	0.00%	96.90%
Tearing of Eyes	0.00%	0.00%	3.20%	0.00%	96.80%
Headaches	0.00%	3.10%	0.00%	0.00%	96.90%
Numbness and Tingling	3.10%	0.00%	3.10%	0.00%	93.80%
Altered Sense of Taste	0.00%	3.10%	3.10%	3.10%	90.60%
Feeling of Fullness	0.00%	0.00%	0.00%	0.00%	0.00%
Muscle Weakness	21.90%	3.10%	3.10%	3.10%	68.80%
Muscle Fatigue	18.80%	3.10%	0.00%	0.00%	78.10%
Tired or Fatigued	34.40%	15.60%	18.80%	6.30%	25.00%

Year 3 Sensory Domain

Year 3 Symptoms	Most Bothersome	Somewhat Bothersome	Less Bothersome	Not Bothersome	Not Reported
Increased Pain	34.40%	9.40%	3.10%	3.10%	50.00%
Diminished Sense of Smell+	3.10%	12.50%	21.90%	15.60%	46.90%
Diminished Sensation	3.10%	6.30%	0.00%	3.10%	87.50%
Double Vision or Vision Changes	0.00%	3.10%	3.10%	6.30%	87.50%
Dry Eyes+	3.10%	0.00%	0.00%	0.00%	96.90%
Tearing of Eyes	3.10%	0.00%	3.10%	0.00%	93.80%
Headaches+	3.10%	6.30%	0.00%	0.00%	90.60%
Numbness and Tingling+	3.10%	6.30%	0.00%	3.10%	87.50%
Altered Sense of Taste+*	0.00%	0.00%	15.60%	15.60%	68.80%
Feeling of Fullness	3.10%	3.10%	0.00%	0.00%	93.80%
Muscle Weakness	25.00%	9.40%	6.30%	3.10%	56.30%
Muscle Fatigue	12.50%	6.30%	6.30%	0.00%	75.00%
Tired or Fatigued	28.10%	18.80%	21.90%	6.30%	25.00%

*Significant Change from prior Year
+Significant Change from baseline(Year 1)

Urinary Domain

Year 1 Urinary Domain

Year 1 Symptoms	Most Bothersome	Somewhat Bothersome	Less Bothersome	Not Bothersome	Not Reported
Urinary Incontinence	0.00%	0.00%	0.00%	0.00%	100.00%
Incomplete Voiding, UTIs, or Other Urinary Problems	0.00%	0.00%	0.00%	0.00%	100.00%
Waking up to go to the Bathroom	31.30%	12.50%	12.50%	12.50%	31.30%
Urinary Frequency or Urgency	12.50%	9.40%	6.30%	6.30%	65.60%

Year 2 Urinary Domain

Year 2 Symptoms	Most Bothersome	Somewhat Bothersome	Less Bothersome	Not Bothersome	Not Reported
Urinary Incontinence	6.30%	3.10%	0.00%	0.00%	90.60%
Incomplete Voiding, UTIs, or Other Urinary Problems	6.30%	0.00%	0.00%	0.00%	93.80%
Waking up to go to the Bathroom*	18.80%	9.40%	6.30%	12.50%	53.10%
Urinary Frequency or Urgency*	15.60%	18.80%	9.40%	3.10%	53.10%

Year 3 Urinary Domain

Year 3 Symptoms	Most Bothersome	Somewhat Bothersome	Less Bothersome	Not Bothersome	Not Reported
Urinary Incontinence+	12.50%	0.00%	3.10%	0.00%	84.40%
Incomplete Voiding, UTIs, or Other Urinary Problems+	6.30%	0.00%	0.00%	0.00%	93.80%
Waking up to go to the Bathroom	15.60%	6.30%	18.80%	21.90%	37.50%
Urinary Frequency or Urgency+	21.90%	18.80%	12.50%	12.50%	34.40%

*Significant Change from prior Year
+Significant Change from baseline(Year 1)

Digestive System Domain

Year 1 Digestive System Domain

Year 1 Symptoms	Most Bothersome	Somewhat Bothersome	Less Bothersome	Not Bothersome	Not Reported
Swallowing or Choking	9.40%	6.30%	0.00%	0.00%	84.40%
Hypersalivation or Drooling	0.00%	6.30%	6.30%	3.10%	84.40%
Dry Mouth	0.00%	0.00%	0.00%	0.00%	100.00%
Throat Clearing or Coughing	0.00%	0.00%	0.00%	0.00%	100.00%
Constipation	12.50%	6.30%	3.10%	6.30%	71.90%
Loss of Appetite	0.00%	3.10%	0.00%	0.00%	96.90%

Year 2 Digestive System Domain

Year 2 Symptoms	Most Bothersome	Somewhat Bothersome	Less Bothersome	Not Bothersome	Not Reported
Swallowing or Choking*	12.50%	9.40%	15.60%	9.40%	53.10%
Hypersalivation or Drooling*	3.10%	3.10%	12.50%	28.10%	53.10%
Dry Mouth	0.00%	3.10%	3.10%	0.00%	93.80%
Throat Clearing or Coughing	0.00%	0.00%	0.00%	3.20%	96.80%
Constipation*	15.60%	9.40%	9.40%	18.80%	46.90%
Loss of Appetite*	0.00%	0.00%	3.10%	0.00%	96.90%

Year 3 Digestive System Domain

Year 3 Symptoms	Most Bothersome	Somewhat Bothersome	Less Bothersome	Not Bothersome	Not Reported
Swallowing or Choking+	9.40%	6.30%	21.90%	15.60%	46.90%
Hypersalivation or Drooling+	0.00%	6.30%	15.60%	21.90%	56.30%
Dry Mouth+	0.00%	0.00%	6.30%	12.50%	81.30%
Throat Clearing or Coughing+	0.00%	6.30%	3.10%	0.00%	90.60%
Constipation**+**	12.50%	9.40%	12.50%	15.60%	50.00%
Loss of Appetite+*	3.10%	3.10%	6.30%	6.30%	81.30%

*Significant Change from prior Year
+Significant Change from baseline(Year 1)

Sexual Domain

Year 1 Sexual Domain

Year 1 Symptoms	Most Bothersome	Somewhat Bothersome	Less Bothersome	Not Bothersome	Not Reported
Impotence or Sexual Dysfunction	3.10%	0.00%	0.00%	0.00%	96.90%

Year 2 Sexual Domain

Year 2 Symptoms	Most Bothersome	Somewhat Bothersome	Less Bothersome	Not Bothersome	Not Reported
Impotence or Sexual Dysfunction	0.00%	3.20%	0.00%	0.00%	96.80%

Year 3 Sexual Domain

Year 3 Symptoms	Most Bothersome	Somewhat Bothersome	Less Bothersome	Not Bothersome	Not Reported
Impotence or Sexual Dysfunction	0.00%	3.10%	0.00%	3.10%	93.80%

*Significant Change from prior Year
+Significant Change from baseline(Year 1)
